# International Homœopathic Congress, 1896

**Published:** 1894-09

**Authors:** 


### INTERNATIONAL HOMŒOPATHIC CONGRESS, 1896.

The Committee appointed by the British Homœopathic Congress of 1891 to organize the Fifth Quinquennial International Homœopathic Congress presents the following recommendations:

1. That the Congress shall assemble in London, at such time and during such number of days as may hereafter be determined.

2. That this meeting take the place of the annual British Congress, and that its officers be elected at the Congress of the preceding year; the International Congress being free to elect Honorary Vice-Presidents from those foreign guests and others whom it desires to honor.

3. That the expenses of the meeting be defrayed by a subscription from the homœopathic practitioners of Great Britain, the approximate amount to be expected from each to be named as the time draws near.

4. That the cost of printing the Transactions be met by a subscription from all who desire to possess a copy of the volume.

5. That the Congress shall be open to all qualified to practice medicine in their own country.

6. That all who attend shall present their names and addresses, and a statement of their qualifications, and, if unknown to the officers of the Congress, shall be introduced by some one known to them, or shall bring letters credential from some Homœopathic Society or other recognized representative of the system.

(*a*) That members of the Congress, as above characterized, shall be at liberty to introduce visitors to the meetings at their discretion.

7. That the Committee be authorized to enter into communication with physicians at home and abroad to obtain—

(*a*) A report from each country supplementary to those presented at previous Quinquennial Congresses, recounting everything of interest in connection with Homœopathy which has occurred within its sphere since its last report was presented.

(*b*) Essays upon the various branches of homœopathic theory and practice, for discussion at the meetings and publication in the Transactions.

8. That all essays must be sent in by January 1st, 1896, and shall then be submitted to a Committee of Censors for approval as suitable for their purpose.

9. That the approved essays shall be printed beforehand, and distributed to such members of the Congress as may apply for them, instead of being read at the meetings.

10. That for discussion the essays shall be presented singly or in groups, according to their subject-matter, a brief analysis of each being given from the chair.

11. That a member of the Congress (or two, where two classes of opinion exist on the subject, as in the question of the dose), be appointed some time before the meeting to open the debate, ten minutes being allowed for such purpose; and that then the essay, or group of essays, be at once opened for discussion, five minutes being the time allotted for each speaker.

12. That the Chairman shall have liberty, if he sees that an essay is being debated at such length as to threaten to exclude later subjects of importance, to close its discussion.

13. That the authors of the essays, if present, shall have the right of saying the last word before the subject is dismissed, ten minutes being granted them for this purpose.

14. That the following circular letter be printed, and sent to all editors of journals, secretaries of societies, and deans of colleges throughout the homœopathic world, soliciting their interest and co-operation.

